# Prostate cancer clinical characteristics and outcomes in Central Sudan

**DOI:** 10.3332/ecancer.2020.1116

**Published:** 2020-10-06

**Authors:** Sami Mahjoub Taha, Hsin-Yi Weng, Mohammed El Imam Mohammed, Yassin M Osman, N’sanh N’dri, Sulma I Mohammed, Dafalla Omer Abuidris

**Affiliations:** 1 Urology Department, Gezira Hospital for Renal Disease and Surgery, PO Box 20, Sudan; 2 Department of Comparative Pathobiology and Purdue University Center for Cancer Research, Purdue University, West Lafayette, IN 47907, USA; 3 Oncology Department, National Cancer Institute, University of Gezira, Sudan

**Keywords:** prostate, cancer, Gleason score, PSA, Sudan, Sudanese

## Abstract

**Background:**

Prostate cancer is the most common cancer among Sudanese men and most patients present at a late stage. Although the incidence of prostate cancer in Sudan is low compared to other African countries, studies on prostate cancer in Sudan are limited. This study addresses the clinical characteristics and outcomes of prostate cancer in Central Sudan and its prognostic factors.

**Methodology:**

This study was conducted prospectively at the Gezira Hospital for Renal Disease and Surgery and at the National Cancer Institute at the University of Gezira, Sudan, for an 11-year period.

**Results:**

During the study period, 543 patients participated in the study. Each one underwent a clinical examination, digital rectal examination and radiological staging using magnetic resonance imaging or computed tomography and provided blood samples for prostate-specific antigen (PSA) testing. The mean (SD) age of patients was 72.6 (9.9) years. At diagnosis, the majority of patients experienced lower urinary tract symptoms (LUTS; 54%), bladder outlet obstructions (OU) without (18%) or with urine retention (14%), PSA median was 100 ng/mL and the mean was 269 ng/mL, locally advanced disease (45%) or distant metastasis (46%). The age-adjusted hazard ratio (HR) of mortality was twofold, comparing patients presented with OU to patients with LUTS. Patients diagnosed with locally advanced and castration resistance prostate cancer had five times the HR compared to patients diagnosed with organ-confined prostate. On the contrary, the HR increased sevenfold for patients with distant metastasis. Gleason score did not show a significant association with survival (*p* = 0.249). Similarly, there was no apparent dose–response association between the PSA levels at diagnosis (*p* = 0.460).

**Conclusion:**

The findings suggest that Sudanese men who are living in Central Sudan present at diagnosis with large tumours at late stages, and high PSA levels and Gleason scores. Improving awareness and building up the treatment capacity are key to achieving better outcomes.

## Introduction

Worldwide, prostate cancer constitutes the fourth most common cancer in both sexes combined and the second most common cancer in men [1]. The prostate cancer incidence rate varies worldwide. According to the Globocan 2012 Report, the incidence rates are highest in Australia/New Zealand, Northern America, and Western and Northern Europe. The incidence rates are also relatively more elevated in less developed areas, such as the Caribbean, Southern Africa and South America, but remain low in Asian populations. The incidence rate in the Caribbean is 29 per 100,000 and in sub-Saharan Africa it is 19–24 per 100,000 [2].

In Sudan, prostate cancer is the most common cancer among Sudanese men and is equally distributed among its different tribes. About 85.4% of these men present with stage III and IV at the mean age of 72.2 ± 9.25 years [3]. Although it is a sporadic disease in men younger than 50 years in Sudan, the rates increase exponentially afterwards. Age, education level and positive family history are the common risk factors associated with prostate cancer in Sudan. Other factors include a history of tobacco and alcohol consumption, body mass index (BMI) and occupation [4].

The research community classifies patients with small size tumours, low prostate-specific antigen (PSA) (<10 ng/mL) and low Gleason scores (<6) as low risk for prostate cancer, whereas patients with larger tumours (T2C and higher) that are growing outside of the prostate, elevated PSA (>20 ng/dl) and high Gleason scores (>8) are classified as high risk for prostate cancer. The 5-year survival rate for the low-risk group is about 100%. About 98% are alive after ten years, and 96% live for at least 15 years after diagnosis. However, men in the high-risk group have a 5-year survival rate of 29% [5].

The age standard rates (ASRs) of prostate cancer among men in Sudan (Khartoum State) were 22.1 (1966 WSP) and 25.2 per 100,000 (2000 WSP) [6]. Data from Harare, Zimbabwe (1991–2010); Kampala, Uganda (1991–2006); Abidjan, Ivory Coast (1995–1997) and Ibadan and Abuja, Nigeria, have indicated higher ASRs for prostate cancer: 73.0, 39.6, 31.4, 17.4 and 25.9 per 100,000 (1966 WSP), respectively [7]. Although the incidence of prostate cancer in Sudan is lower compared to other African countries, there are limited studies on prostate cancer in Sudan. In this study, we aimed to characterise prostate cancer in Central Sudan by describing the symptoms at presentation, risk, age distribution, treatment, PSA and Gleason score among prostate cancer patients by relating them to survival in order to identify potential prognostic factors.

## Methods

### Setting

The study was conducted retrospectively at the Gezira Hospital for Renal Disease and Surgery (GHRDS) and at the National Cancer Institute at the University of Gezira (UGNCI) from 2005 to 2016. The GHRDS provided diagnosis and treatment interventions to prostate cancer patients, whereas the UGNCI provided hormonal and radiological therapies.

### Study population

The study population consisted of patients diagnosed with histopathology confirmed prostate cancer during the study period at GHRDS. Also, the study included patients who visited GHRDS for treatment (such as subcapsular orchiectomy). Also, we collected data from patients registered at the UGNCI who came for radiotherapy treatment, and patients who came for follow-up to the onco-urology combined clinics.

### Variables and data collection

The variables assessed were age at diagnosis, clinical presentation, cancer stage or risk at diagnosis, tumour classification, treatment, PSA level and Gleason score. We recorded the clinical presentations of the patients at the initial visits, which included unilateral or bilateral ureteral obstruction, urine retention, bladder outlet obstruction and lower urinary tract symptoms (LUTS). The cancer stage of the patients (I–IV) at diagnosis was determined at the clinical stage, by using a digital rectal examination, and at the radiological stage, by using computed tomography (CT). The patients were classified according to the radiological stage using magnetic resonance imaging (MRI) or CT (if MRI was unavailable). We classified the tumours as organ-confined (low risk), locally advanced, distant metastasis (high risk) and castration-resistant. The PSA levels (ng/mL) at diagnosis was grouped into <4, 4–20, 21–50, 51–100, 101–200 and >200. Gleason score, ranging from 2 to 10, was grouped into 2–6, 7 and 8–10. We determined the vital status (e.g., alive or dead) of the patients by follow-up phone calls or death certificates for those who did not show up and by recording the date of death, respectively. We also recorded the date of last contact (e.g., hospital visit or follow-up phone calls) for patients who were alive during the follow-up period (i.e., censoring). Patients who could not be reached by phone calls or did not have records after the initial cancer diagnosis and treatment were not included in this study.

### Treatment guidelines

Cases having early disease (I/II) are supposed to undergo radical surgery or radiation therapy, provided their life expectancy is more than 10 years. However, there is not enough experience in performing radical prostatectomy among urologists, probably due to the small number of cases presenting at early stages.

Radical radiotherapy (dose beyond 80 Gy) cannot be conducted in Sudan due to the shortage of treatment machines, long waiting time (4 months) and type of machines (only cobalt 60). Most patients end up having hormonal treatment in the form of bilateral orchidectomy, followed by antiandrogens when biochemical failure occurrs and if the symptoms progress. Antiandrogens in the form of biclutanide are provided for free to all patients up to 2 years when castration resistance starts to develop in most cases. We continue with the antiandrogen therapy, after castration resistance, along with other treatments like chemotherapy or abiraterone acetate or enzalutamide; the latter two drugs are usually available for cases who can pay for them from their own pockets.

### Ethical approval

The patients consented verbally. The ethical committee at the Faculty of Medicine, the research committee at the Gezira Hospital for Renal Disease and Surgery and the National Cancer Institute, University of Gezira, approved this study.

### Data analysis

We analysed the data using IBM SPSS Statistics (Version 23. Armonk, NY: IBM Crop) to characterise the distribution of study variables in the study population. We assessed the prognostic factors using Cox’s proportional hazards regression and Kaplan–Meier’s survival analyses. Age-adjusted hazard ratios (HRs) and corresponding 95% confidence intervals (CIs) were reported. Statistical significance was set at *p* < 0.05.

## Results

The study included a total of 583 patients with prostate cancer diagnosis during the period from January 2005 to December 2016 at the participating hospitals. We successfully followed-up 453 patients after the initial cancer diagnosis and treatment. The mean (SD) age at diagnosis of the study participants was 72.6 (9.9) years. Patients resided in 16 different counties in Central Sudan. Most of the patients (72%) presented with stage IV and (27%) were at stage III, while only 1.1% presented at stage I/II at diagnosis (Table 1). The majority of the patients (54%) initially presented with LUTS, which included voiding or irritative voiding, bladder outlet obstruction (18%) and urine retention (14%). Furthermore, the majority of the patients had metastatic (46%) or locally advanced (45%) diseases (Table 1) (high-risk group). Hormonal therapy was the most common treatment (70%) administered to the study participants. The median (range) length of follow-up was 24 (0–79) months. During the follow-up, 202 (45%) patients died. The overall 5-year survival rate was 43%. The 5-year survival rate for patients with stage III diagnosis was 57% and was 40% for stage IV patients. The 5-year survival rates for patients with low risk, locally confined or high-risk advanced prostate cancer were 88% and 56%, respectively. The 5-year survival rates decreased to 37% and 30% in patients with distant metastasis and castration resistance (clinical or biochemical progression with testosterone levels less than 50 ng/mL), respectively. Table 2 summarises the results of Cox’s proportional hazards regression.

**Figure 1.**
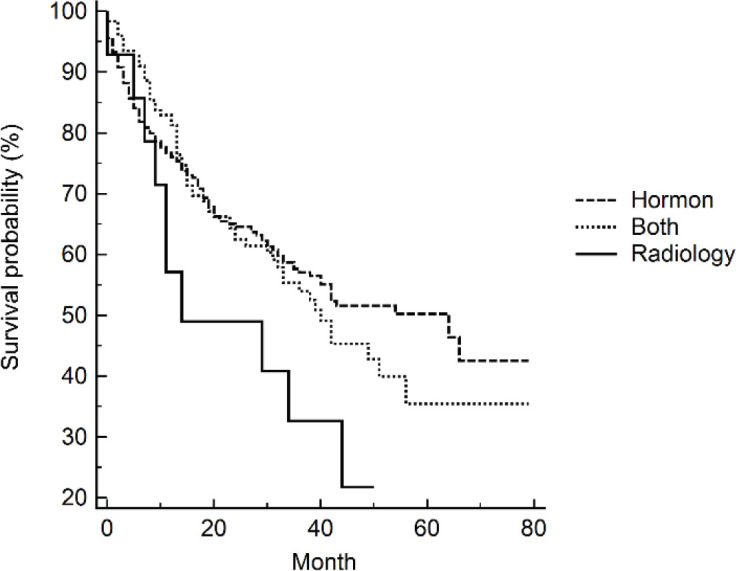
Kaplan–Meier’s survival curves by treatments.

Clinical presentation and tumour classification were significant predictors of prognosis. Patients presented with unilateral or bilateral ureteric invasion and obstruction had two times the rate of dying compared to patients with LUTS. The median survival time (95% CI) was 19 (3–35) months and 15 (0.4–30) months for patients with unilateral or bilateral ureteric, respectively. Patients diagnosed with locally advanced and castration resistance prostate cancer had five times the rate of dying compared to patients diagnosed with organ-confined prostate. On the contrary, the rate increased to sevenfold for patients with distant metastasis. The median survival time (95% CI) was 34 (27–41) months for patients with distant metastasis. Neither Gleason score (*p* = 0.249) nor PSA (*p* = 0.460) level showed a significant dose–response relationship with survival after adjusting for age, although both showed increased HRs compared to the reference category. Patients who received radiology-only therapy had the worst prognosis compared to those who received hormone-only therapy or hormone–radiology combined therapy, although the differences were not significant. The median survival time (95% CI) for patients receiving hormone-only therapy or hormone–radiology combined therapy was 64 months (44–84) and 40 months (27–53), respectively, compared to 14 months (0–43) for patients receiving radiology-only therapy (Figure 1).

## Discussion

Sudan was the largest country in Africa until 2011 when South Sudan separated into an independent country [8]. Sudan’s population is highly diverse, consisting of approximately 19 different ethnic groups and 600 subgroups [8]. Political and economic instabilities affect the Sudanese health system negatively with devastating outcomes. Currently, cancer is commonly diagnosed and often claims the lives of many people in Sudan. Prior studies from the Sudan National Cancer Registry indicate that the most common cancer sites in Sudanese men were prostate, leukemia, lymphoma, oral, colorectal and liver [6]. We have previously reported that prostate cancer was identified as the most common cancer in Sudanese men [3].

In this study, we further examined the characteristics of patients diagnosed with prostate cancer in two health institutions in Central Sudan. We found that the mean age at diagnosis of the participants was 72.6 years, which was older compared to a previous study carried out in the Western Cape region of South Africa (69.7 years (*n* = 291) in Whites and 68.9 years in regionally matched Blacks (*n* = 71)) [9]. Similarly, the mean age of prostate cancer at presentation found in this study was slightly older than that reported in another South African prostate cancer study (71.0 years) [10] and more closely similar to that reported in Uganda (*n* = 210; 70.6 years) [10]. The older age at diagnosis found in this study suggests that either prostate cancer was more likely to affect older men or it was due to the lack of cancer screening and awareness of prostate cancer and its symptoms among Sudanese men of all ages. This lack of awareness is reflected in the stage at which these patients presented at diagnosis as most patients present at advanced local or late high-risk metastatic disease. Most patients pursued help only when they experienced lower or other urinary tract symptoms. This late clinical presentation of prostate cancer with advanced metastatic disease is common in all sub-Saharan African countries as these countries lack established systematic screening practices, using PSA or digital rectal examinations for prostate cancer screening, compared to that in the developed world where PSA and other screening modalities are common for early prostate cancer detection. In the developed countries, 90% of men are diagnosed with a confined disease, which has a 100% survival rate.

The PSA level correlates with a higher likelihood of prostate cancer diagnosis and death in younger men. However, there are varying controversies among clinicians regarding the usefulness of PSA, as research findings have indicated that the test leads to overtreatment for detecting slow-growing prostate cancer lesions. Also, PSA testing in older men has limited utility [11], which is in agreement with our study. In our study, 92% of the patients had >4.0 ng/mL of PSA because the majority of men had advanced prostate cancer. Therefore, our study found no dose–response association between the PSA levels at diagnosis and prognosis (*p* = 0.460). The high PSA levels in Sudanese prostate cancer patients are in agreement with PSA levels reported previously in prostate cancer patients in other African countries [12] and is pertinent again to the fact that most men already had advanced disease.

In this study, we found that the clinical presentation and tumour classification were significant predictors of prognosis. The Gleason score is one of the most widely used and accepted histopathological method for providing information about the prognosis of prostate cancer [13]. In this study, most patients are diagnosed with stage III and IV, high PSA level and high Gleason score. In this study, the Gleason score was an excellent prognostic predictor; however, it did not show a dose–response relationship with survival. This finding was not surprising as poorly differentiated prostate cancer is commonly reported in African men [14,15]

Most of the patients in this study presented with late presentations, and as a consequence, they end up having palliative treatment. Patients with relatively early disease were treated with hormonal therapy and radiotherapy dose of 60 Gy or less, which is not enough to treat prostate cancer radically (only cobalt machines are available and no 3D conformal treatment). Several clinical trials have demonstrated the importance of dose escalation for improved control of high-risk prostate cancer [16]. The suboptimal dose reflects the inadequate capacity of radiotherapy service in Sudan, which is common in sub-Saharan Africa [17].

Early diagnosis of prostate cancer and treatment in Sudan faces many obstacles such as the absence of multiparametric MRI, erratic function of the trans-rectal ultrasound machine and the discrepancy of PSA laboratory results. Another challenge that hampers the successful treatment of patients with prostate cancer in Sudan is that radical surgery is not common practice in Sudan due to the lack of experienced surgeons and the late advanced disease at presentation. The available modalities for treating prostate cancer in Sudan are radiotherapy and hormonal therapy. Hormonal therapy includes LHRH-analogs and antiandrogens.

There were some limitations encountered in our study. The major ones, beside being retrospective, include the small number of patients, the fact that patients have late-stage prostate cancer and the short follow-up time for some of the studied cases because of their immediate death after diagnosis. However, despite that, this is the first study to report the clinical presentation and utility of PSA level and Gleason score as a predictor of prostate cancer prognosis in Sudanese men.

## Conclusion

This study describes the clinical features of prostate cancer in men living in Central Sudan. Men living in Central Sudan present at diagnosis with large tumours, late disease stages, high PSA levels and high Gleason scores. These features represent a lack of awareness among Sudanese men in Central Sudan. Therefore, the need for more studies exists to address why African men are often diagnosed with poorly differentiated prostate cancer and whether this is a result of the environment, genetics or due to a lack of screening and awareness. This study addresses the issue of improving community awareness about this disease in order to change the fate of cases. This change will not be achieved without building up our capacity in treatment machines and training to perform radical prostate cancer surgery.

## Conflicts of interest

The authors declare no conflicts of interest.

## Funding statement

No funding was provided for this study.

## Authors’ contributions

Sami Mahjoub Taha, Mohamed El Imam Mohamed Ahmed, Yassin M Osman and Dafallah abudiris conceptualised the study and the design. Sami Mahjoub Taha, Mohamed El Imam Mohamed Ahmed, Yassin M Osman, Hsin-Yi Weng N’sanh N’dri and Sulma I Mohammed assisted with the data acquisition and analysis. Hsin-Yi Weng N’sanh N’dri and Sulma I Mohammed drafted the manuscript. Sami Mahjoub Taha, Mohamed El Imam Mohamed Ahmed, Yassin M Osman, Dafallah abudiris, Hsin-Yi Weng N’sanh N’dri and Sulma I Mohammed approved the final manuscript.

## Figures and Tables

**Table 1.** Distribution of the characteristics of prostate cancer in patients presented at a participating hospital in Central Sudan (*n* = 453).

Characteristics	Distribution
Stage at diagnosis	
I	2 (0.4%)
II	3 (0.7%)
III	122 (27%)
IV	326 (72%)
Primary clinical presentation	
Lower urinary tract symptoms	243 (54%)
Bladder outlet obstruction	82 (18%)
Urine retention	61 (14%)
Unilateral ureteral obstruction	21 (5%)
Bilateral ureteral obstruction	20 (4%)
Others	26 (6%)
Classification	
Organ confined	9 (2%)
Locally advanced	205 (45%)
Castration resistance	31 (7%)
Distant metastasis	208 (46%)
Gleason score	
2–6	67 (16%)
7	113 (27%)
8–10	244 (58%)
Prostate-specific antigen level at diagnosis (ng/mL)	
<4	40 (9%)
4–20	62 (14%)
21–50	53 (12%)
51–100	61 (14%)
101–200	162 (36%)
>200	75 (17%)
Treatment^a^	
Hormonal only	315 (70%)
Radiotherapy only	14 (3%)
Hormonal + Radiotherapy	123 (27%)
Vital status at the end of follow-up^b^	
Alive	251 (55%)
Dead	202 (45%)

a One patient reported that chemotherapy was excluded.

b Length of follow-up ranging from 0 to 79 months, with a median of 24 months.

**Table 2.** Age-adjusted hazard ratios of death by the characteristics of prostate cancer in patients presented at a participating hospital in Central Sudan (n = 453).

Characteristics	Hazard ratio (95% confidence interval)	*p*-value
Primary clinical presentation		
Lower urinary tract symptoms	Reference	0.017
Bladder outlet obstruction	1.2 (0.8–1.7)	
Urine retention	1.5 (1.0–2.2)	
Unilateral ureteral obstruction	2.1 (1.2–3.6)	
Bilateral ureteral obstruction	2.1 (1.2–3.8)	
Others	1.6 (0.9–2.8)	
Classification		
Organ confined	Reference	0.017
Locally advanced	4.9 (0.7–35.2)	
Castration resistance	5.3 (0.7–40.6)	
Distant metastasis	7.2 (1.0–51.7)	
Gleason score		
2–6	Reference	0.249
7	1.5 (0.9–2.4)	
8–10	1.2 (0.8–1.9)	
Prostate-specific antigen level at diagnosis (ng/mL)		
<4	Reference	0.460
4–20	1.3 (0.6–2.8)	
21–50	1.4 (0.6–3.0)	
51–100	1.3 (0.6–2.7)	
101–200	1.7 (0.9–3.5)	
>200	1.4 (0.6–2.8)	
Treatment^a^		
Hormonal only	Reference	0.121
Radiotherapy only	1.9 (1.0–3.7)	
Hormonal + Radiotherapy	1.1 (0.8–1.5)	

a One patient reported that chemotherapy was excluded.
